# From contemplation to serenity: how yoga meditation improves the mental health of female college students?

**DOI:** 10.3389/fpsyg.2025.1545943

**Published:** 2025-03-10

**Authors:** Lanjuan Liu, Cheng Liu, Lijun Tang, Xing Wang, Qiangming Feng

**Affiliations:** ^1^School of Physical Education, Shanghai Normal University, Shanghai, China; ^2^Department of Physical Education, Donghua University, Shanghai, China; ^3^Department of Physical Education, Shanghai Southwest Weiyu Affiliated Experimental School, Shanghai, China; ^4^Department of Physical Education, Shanghai International Studies University, Shanghai, China

**Keywords:** yoga meditation, mental health, emotional regulation, stress relief, psychological resilience

## Abstract

**Objective:**

This study aims to investigate the impact of yoga meditation on the mental health of female college students, focusing on how meditation improves emotional regulation, alleviates stress and strengthens psychological resilience.

**Methods:**

Employing a combination of quantitative assessment and qualitative analysis, the study measured participants’ emotional states, stress levels, and psychological resilience across multiple time points to track participants’ mental health changes dynamically. In-depth interviews and analysis of meditation journals were also conducted.

**Results:**

Yoga meditation significantly reduced anxiety, depression, and perceived stress while enhancing emotional regulation and self-awareness. Meditation positively influenced neuroplasticity, inducing beneficial changes in brain regions associated with emotional control and cognitive flexibility. Additionally, improved autonomic nervous system function was observed, with increased parasympathetic activity and reduced sympathetic response. Meditation strengthened psychological resilience in female college students, improved stress-coping strategies, and sustained positive mental health benefits even after the intervention.

**Conclusion:**

Yoga meditation is an effective mental health intervention, bolstering emotional regulation and reducing stress among female college students. Integrating yoga meditation into campus mental health programs is recommended to provide students with greater practice opportunities and personalized guidance.

## 1 Introduction

With the rapid evolution of social and educational environments, female college students face unprecedented psychological pressures and challenges. Academic demands, career competition, and the complexities of social relationships often overwhelm students regarding emotional regulation and psychological coping. Compared to other demographics, female college students report higher incidences of anxiety, depression, and stress-related issues (Jones et al., 2022). These mental health challenges not only impact academic performance and quality of life but also have profound negative effects on long-term psychological well-being. Consequently, identifying effective interventions to help female college students regulate emotions and build psychological resilience has become a focal point in psychological research.

Among various exercise-based interventions, yoga meditation has garnered academic attention due to its unique combination of physical activity and mental discipline. Yoga meditation incorporates mindful breathing, body control, and mindful awareness practices to enhance self-awareness and emotional regulation (Jones et al., 2023). Research indicates that yoga meditation effectively alleviates anxiety, depression, and stress, positively impacting subjective well-being and psychological resilience (McLafferty et al., 2021; Liu et al., 2024). Its core mechanism involves modifying individuals’ cognitive responses to external stressors, helping them achieve emotional balance and inner peace (Jones et al., 2021). While the efficacy of yoga meditation has been validated in the general population, systematic studies specifically targeting female college students remain limited, particularly regarding the underlying mechanisms through which meditation improves mental health.

The mental health needs of female college students are distinct. Compared to male students, they tend to adopt emotion-focused stress coping strategies rather than problem-focused approaches, making them more prone to emotional fluctuations and psychological stress (Matud, 2004). Furthermore, lower emotional regulation capabilities and relatively weaker psychological resilience further exacerbate their mental vulnerability (Vigo et al., 2021). Although preliminary research has explored the impact of yoga meditation on this demographic, there is still a lack of dynamic tracking of the psychological changes, especially in understanding the transition from anxiety and stress to inner peace. Thus, a deeper investigation into the dynamic role of yoga meditation in enhancing mental health among female college students holds substantial theoretical and practical significance. This study focuses on the specific improvement processes in mental health facilitated by yoga meditation, particularly regarding emotional fluctuation, stress management, and resilience enhancement. Employing a combination of quantitative and qualitative methods, we dynamically track the influence of meditation on the psychological state of female college students, aiming to elucidate the process by which meditation practice transitions from initial psychological distress to inner peace. This research seeks to answer the following key questions: (1) Does yoga meditation improve mental health among female college students? (2) During meditation practice, does the psychological state of female college students reflect a dynamic shift from anxiety and stress to inner peace? (3) Which psychological mechanisms play key roles in this process? Systematically addressing these questions will provide new empirical evidence for the use of yoga meditation as a mental health intervention for female college students and establish a theoretical and empirical foundation for promoting this method in broader fitness populations.

## 2 Participants and methods

### 2.1 Participants

This study recruited sophomore female college students from a university in Shanghai city using a convenience sampling method. An initial 86 participants were screened and enrolled in a 16-week experimental intervention. Following strict inclusion and exclusion criteria, 80 eligible female students were formally included and randomly assigned to an experimental group (*n* = 40) or a control group (*n* = 40).

**Inclusion criteria:** (1) Experimental group: sophomore female students; voluntary participation with informed consent; full attendance in group yoga sessions; cumulative self-meditation time of at least 400 min; completion of emotional diary entries for meditation practice; participation in group interviews after each intervention phase. (2) Control group: sophomore female students; voluntary participation with informed consent; no involvement in yoga or meditation interventions, maintaining their usual lifestyle.

**Exclusion criteria:** prior participation in similar yoga or meditation training within the last six months; the presence of physical or psychological conditions that may hinder study participation, such as severe mental disorders or physical disabilities; in the experimental group, missing one or more group yoga sessions or cumulative self-meditation time below 400 min during the intervention period. The information about the two groups is shown in Table 1.

**Table 1:** Participant demographics.

Participant ID	Gender	Major	Meditation duration (minutes)
A1	Female	Education	1,323
A2	Female	Literature	1,142
A3	Female	Engineering	1,134
A4	Female	Science	1,131
A5	Female	Law	1,129
A6	Female	Education	1,126
…	…	…	…
A79	Female	Literature	0
A80	Female	Management	0

### 2.2 Literature review

A combination of database searches and manual retrieval was employed to collect a comprehensive set of Chinese and English literature related to the mental health of female college students, yoga meditation interventions, and the associated mechanisms and effects. The literature review focused on several key areas: the mental health status of female college students and its influencing factors, the effectiveness of yoga meditation interventions on mental health, including case studies, theoretical discussions and empirical studies on the mechanisms of yoga meditation interventions, and the selection and application of mental health assessment tools. A systematic analysis of these studies was conducted to summarize existing research findings, identify current hot topics and points of debate, and outline the theoretical framework and practical pathways for evaluating the effectiveness of yoga meditation in mental health interventions.

### 2.3 Expert interviews

In-depth interviews with experts in related fields were conducted to gather professional insights and experiences concerning the mental health of female college students and yoga meditation interventions. These discussions focused on emerging trends and challenges in mental health intervention research, such as capturing changes in mental health status in dynamic environments, addressing cultural differences in mental health perceptions, and effectively enhancing individual mental health through intervention programs. The experimental intervention program was refined and finalized based on the literature review and expert interviews.

### 2.4 Arrangement of yoga and meditation intervention

**Figure 1:**
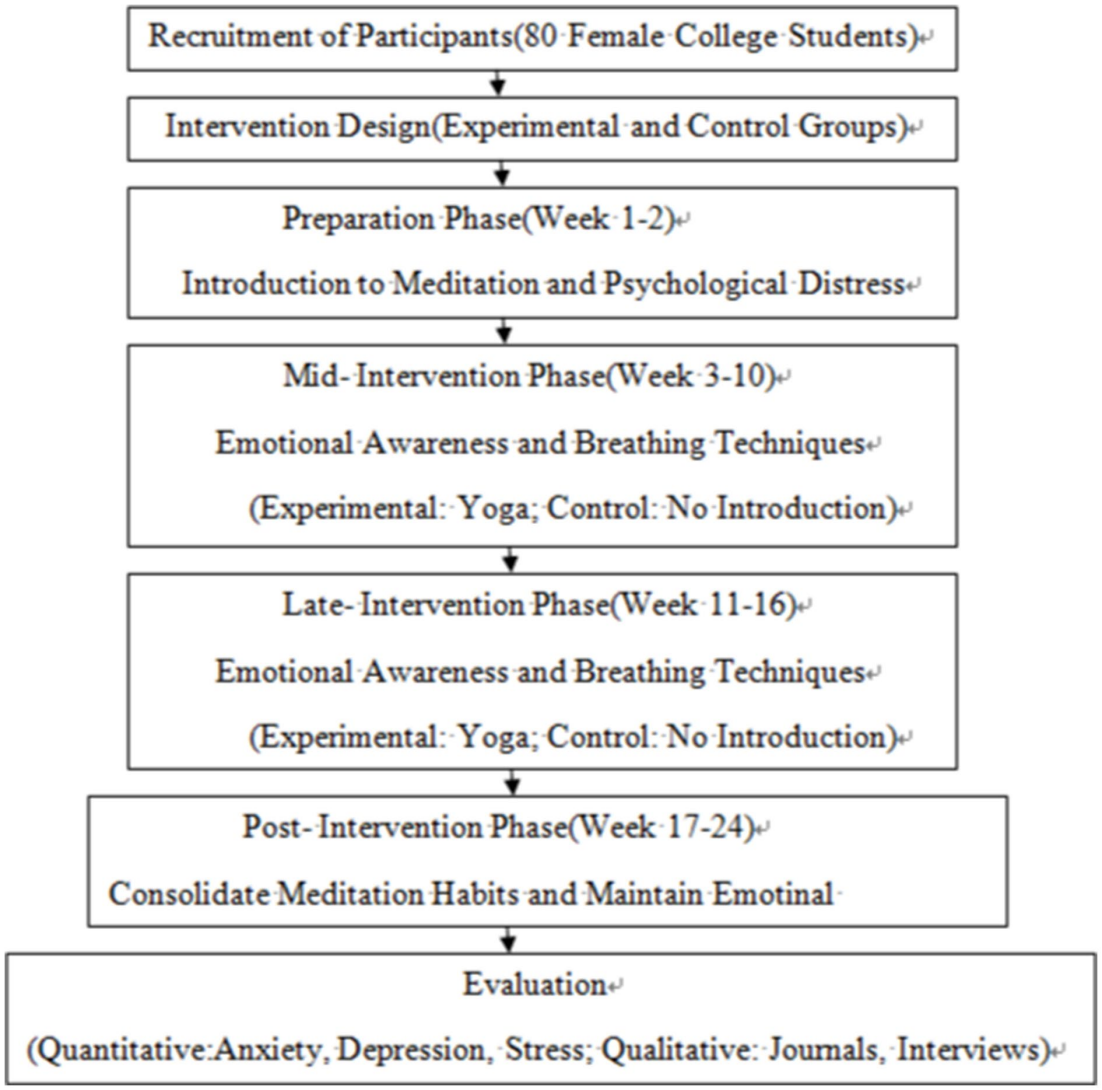
Yoga meditation intervention process for enhancing mental health in female college students.

As shown in Figure 1, the intervention process is divided into several stages, including the preparation, intervention, and post-intervention phases.

Participants were divided into two groups: experimental and control groups. The intervention consisted of 16 weeks of yoga meditation practice for the experimental group, while the control group received no exercise intervention. Self-Rating Anxiety Scale (SAS), Self-Rating Depression Scale (SDS), Perceived Stress Scale (PSS), Connor-Davidson Resilience Scale (CD-RISC), and Emotion Regulation Questionnaire (ERQ) were assessed during and after the intervention. The experimental intervention protocol was as follows.

### 2.5 Composition of yoga meditation intervention group

The intervention team included one graduate instructor (a professional yoga instructor with 13 years of university teaching experience), one graduate student, and three class monitors (undergraduate students).

### 2.6 Design of intervention materials

An interview outline was developed to gather data on participants’ mental states at different stages, their perceptions of mental health, emotional recognition, understanding of yoga meditation, and meditation and mental health practices. The intervention program involved collective meditation sessions, group meditation intervention according to different stages, self-guided use of the “Meditation Planet” app, group discussions, follow-up interviews, and continuous monitoring through the WeChat platform. Intervention measures were tailored to different stages to address specific needs.

#### 2.6.1 On-campus group intervention

Conducted by a professional yoga instructor, group sessions involved groups of 20 students and were held once a week for 90 min each. The first two weeks were a preparation phase, focusing on basic yoga knowledge, breathing regulation, and body awareness exercises to familiarize participants with fundamental concepts and techniques. Weeks 3–16 comprised the meditation intervention phase, including yoga posture practice, breath control, and mindfulness meditation. The curriculum progressively advanced, integrating breathing regulation, body scans, and relaxation techniques to enhance emotional regulation and self-awareness.

#### 2.6.2 Self-guided meditation via app

Participants used the “Meditation Planet” app, covering stress management, emotional regulation, personal growth, relationship improvement, and self-identity. They engaged in self-practice sessions of approximately 10 min each, at least four times weekly, over the 16 weeks. During the intervention, participants were encouraged to choose appropriate meditation audio based on the causes of emotional fluctuations and engage in deep emotional exploration in a safe, distraction-free environment. They recorded emotional changes and practice experiences after each session. After the group intervention, participants continued with self-guided meditation for an additional eight weeks, practicing at their frequency and recording their experiences. This phase aimed to evaluate the sustained effect of meditation on participants’ self-regulated emotional adjustment and long-term intervention outcomes, as shown in Table 2.

**Table 2:** Overview of intervention measures at different stages.

Intervention phase	Duration	Objective	Intervention content
Preparation phase (T1)	Weeks 1–2	Recognize psychological responses, grasp basic meditation knowledge, and learn to use the “Meditation Planet” app.	Understand basic concepts and benefits of meditation, recognize psychological distress, practice with the “Meditation Planet” app, and read related books.
Mid-intervention phase (T2)	Weeks 3–10	Master breathing techniques and recognize emotional fluctuations. Enhance self-awareness	Practice breathing relaxation, muscle relaxation, and body scans. Recognize emotional fluctuations and improve emotional awareness.
Late-intervention phase (T3)	Weeks 11–16	Learn emotion management techniques; understand emotion regulation strategies and psychological responses.	Practice techniques such as breath regulation, diaphragmatic breathing and alternate nostril breathing to enhance emotional regulation and resilience
Post-intervention phase (T4)	Weeks 17–24	Consolidate meditation habits; maintain long-term inner peace and emotional regulation.	Continue using meditation techniques for daily emotion management, record psychological changes, reflect regularly, and sustain meditation habits.

#### 2.6.3 Research design

This study utilized a convergent mixed-methods design, collecting quantitative data through SAS, SDS, PSS, CD-RISC, and ERQ during and after the intervention. Qualitative data were collected through semi-structured interviews and meditation journals. The results from both data types were compared and integrated to understand the research questions comprehensively.

#### 2.6.4 Quality control of the experiment

To ensure participants completed the intervention effectively, the following quality control measures were implemented: (a) A WeChat group was established to encourage students to share practice photos, facilitating exchanges of experiences among peers and between students and teachers; (b) The instructor provided real-time responses to queries to maintain engagement; (c) Attendance and experience sharing were included in the course assessment, employing motivational strategies to increase student adherence.

### 2.7 Qualitative analysis

Group-based, semi-structured interviews were conducted to capture participants’ genuine experiences with meditation practice, with audio recording and note-taking upon participants’ informed consent. Each interview, conducted by the same interviewer, lasted approximately one hour. Interview content covered understanding of emotional states, experiences with meditation, issues encountered, changes in physical and mental states, and the impact of meditation on negative emotions, emphasizing participants’ descriptions of their experiences. Participants’ self-guided meditation logs were collected, and recordings were transcribed. These transcriptions and meditation logs were imported into Nvivo12 software for qualitative analysis, following steps such as repeated reading, initial theme identification and coding, and refinement of primary themes. Two researchers performed the coding process, achieving an 84% inter-coder agreement rate. Discrepancies were resolved through joint discussion to maximize alignment between coded content and themes.

### 2.8 Questionnaire method

Self-Rating Anxiety Scale (SAS): Developed by Zung (1971), this scale includes 20 items rated on a 4-point scale (1 to 4) to assess participants’ anxiety over the past week. The raw score is multiplied by 1.25 to obtain a standard score (25 to 100), with higher scores indicating higher anxiety levels. The internal consistency reliability (Cronbach’s *α*) of the SAS is approximately 0.81 (Zung, 1971).

Self-Rating Depression Scale (SDS): Created by Zung et al. in 1965, the SDS evaluates the severity of depressive symptoms (Zung, 1965). This 20-item scale also uses a 4-point rating () to reflect depressive states over the prior week. The standard score (25 to 100) is derived by multiplying the raw score by 1.25. The SDS demonstrates an internal consistency reliability (Cronbach’s *α*) of 0.84 (Zung, 1965).

Perceived Stress Scale (PSS): Developed by Cohen et al. in 1983, the PSS measures subjective perceptions of stress (Cohen et al., 1983). The scale consists of 10 items rated on a 5-point scale (0 to 4), with a score range of 0 to 40, where higher scores reflect greater perceived stress. The internal consistency reliability (Cronbach’s *α*) of the PSS is around 0.78 (Cohen et al., 1983).

Connor-Davidson Resilience Scale (CD-RISC): Created by Connor and Davidson in 2003, the CD-RISC assesses an individual’s psychological resilience in the face of stress and adversity (Connor and Davidson, 2003). The scale comprises 25 items rated on a 5-point scale (0 to 4), with a total score range of 0 to 100, where higher scores indicate greater resilience. This scale has shown high reliability and validity across diverse cultural contexts (Cronbach’s *α* approximately 0.89) (Connor and Davidson, 2003).

Emotion Regulation Questionnaire (ERQ): Developed by Gross and John in 2003, the ERQ assesses cognitive reappraisal and expressive suppression strategies in emotion regulation (Gross and John, 2003). The scale consists of 10 items rated on a 7-point scale (), with higher scores indicating more frequent use of these strategies. The internal consistency reliability (Cronbach’s *α*) of the ERQ is 0.82 for cognitive reappraisal and 0.76 for expressive suppression (Gross and John, 2003).

### 2.9 Statistical analysis

Data analysis was conducted using SPSS and AMOS software. Descriptive statistics were used to outline the basic characteristics of the participants, and an independent samples t-test and chi-square test assessed baseline (T1) homogeneity between the experimental and control groups. Repeated measures analysis of variance (ANOVA) was then employed to examine changes in mental health indicators across different time points, exploring the main and interaction effects of yoga meditation on psychological states. Structural Equation Modeling (SEM) was used to analyze the potential mediating effects of meditation on mental health outcomes, such as the role of emotional regulation in reducing anxiety and coping with stress. In addition, qualitative analysis of the meditation experiences in the experimental group employed thematic analysis to extract participants’ subjective perceptions of emotional regulation and inner calm, offering a comprehensive understanding of the dynamic mechanisms of meditation intervention.

## 3 Results and analysis

### 3.1 Descriptive statistics and homogeneity test

Eighty female college students meeting the study criteria were recruited and randomly assigned to the experimental and control groups, with 40 participants in each group. Baseline (T1) descriptive statistics and homogeneity testing were conducted for the two groups. The results indicated no statistically significant differences in age or baseline mental health indicators, including anxiety, depressive symptoms, perceived stress, psychological resilience, and emotional regulation abilities between the experimental and control groups (*p* > 0.05) (Table 3). The mean values and standard deviations for each variable were comparable, with all *p*-values exceeding 0.05 in the independent samples t-test, demonstrating no significant differences between groups on these baseline characteristics (Table 3).

**Table 3:** Descriptive statistics and group homogeneity test.

Variable	Experimental group	Control group	*T*	*P*
Age	20.1 ± 1.2	20.3 ± 1.1	0.56	0.578
SAS	52.4 ± 6.8	51.9 ± 7.0	0.48	0.632
SDS	56.2 ± 7.5	55.8 ± 6.9	0.36	0.719
PSS	29.8 ± 4.9	29.2 ± 5.1	0.74	0.465
CD-RISC	62.4 ± 8.1	63.0 ± 7.8	0.58	0.563
ERQ	25.6 ± 4.7	26.0 ± 4.5	0.49	0.623

SAS, self-rating anxiety scale; SDS, self-rating depression scale; PSS, perceived stress scale; CD-RISC, Connor-Davidson resilience scale; ERQ, emotion regulation questionnaire.

### 3.2 Repeated measures ANOVA

As shown in Table 4, the experimental group demonstrated significant improvements across all five mental health dimensions, whereas no notable changes were observed in the control group. Anxiety, depression, and perceived stress levels in the experimental group showed a significant decrease, while psychological resilience and emotional regulation abilities improved significantly. These changes were sustained after the intervention concluded. All group × time interaction effects were significant, indicating the notable effectiveness of the yoga meditation intervention in enhancing mental health, with no comparable changes observed in the control group.

**Table 4:** Descriptive statistics of five scales for experimental and control groups.

Variable	Experimental group T1	Experimental group T2	Experimental group T3	Experimental group T4	Control group T1	Control group T2	Control group T3	Control group T4
SAS	51.513	46.373	44.233	41.939	51.863	48.779	49.873	51.068
SDS	57.474	51.187	49.064	44.565	54.734	54.834	51.512	51.265
PSS	30.269	26.484	24.297	22.859	29.898	29.280	27.574	25.384
CD-RISC	61.362	64.571	69.890	72.152	62.504	64.349	65.831	63.992
EQR	24.370	27.806	31.914	31.982	25.974	27.030	26.434	25.049

SAS, self-rating anxiety scale; SDS, self-rating depression scale; PSS, perceived stress scale; CD-RISC, Connor-Davidson resilience scale; ERQ, emotion regulation questionnaire.

### 3.3 Structural equation modeling (SEM) analysis

**Figure 2:**
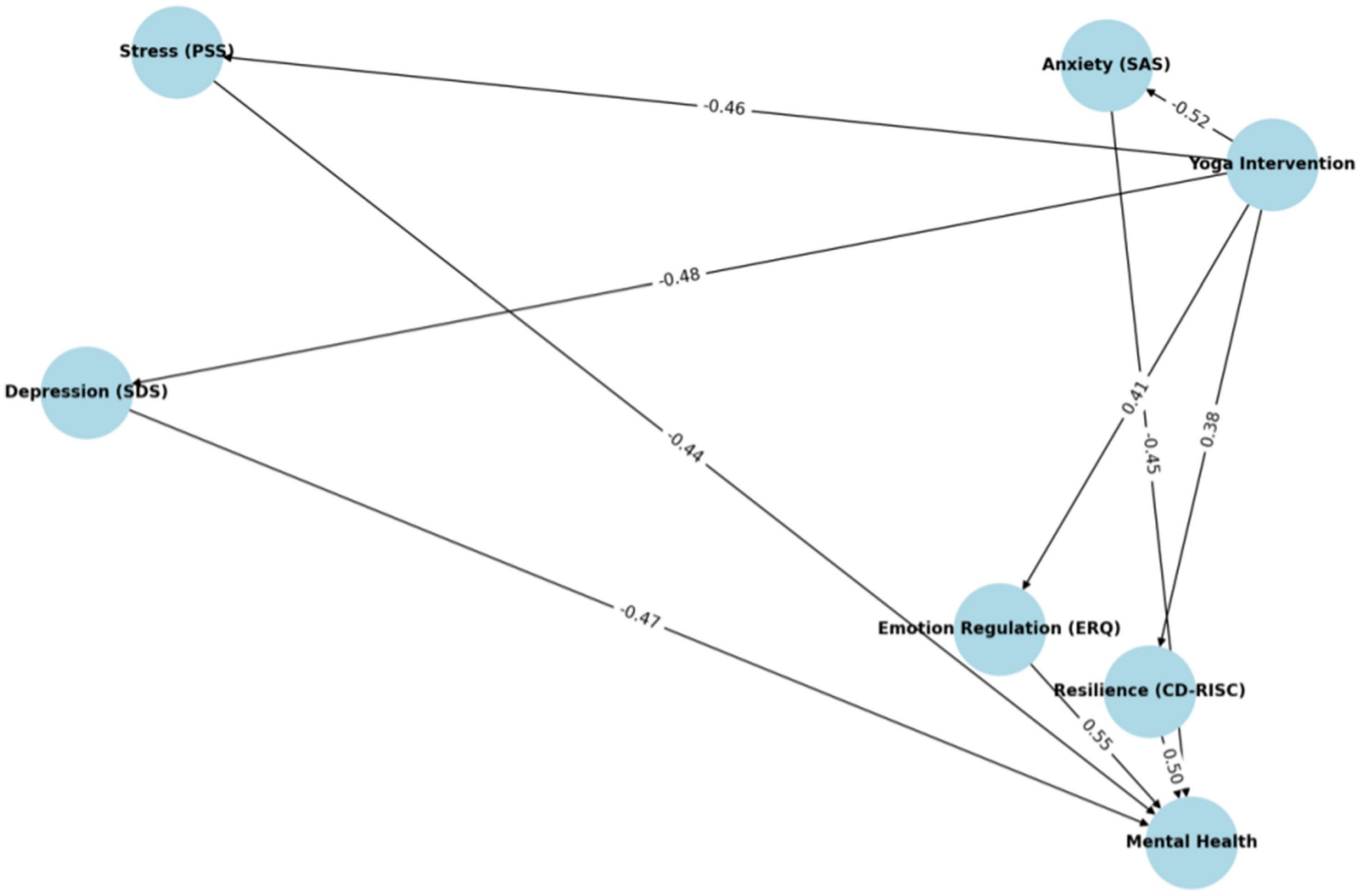
Structural equation model of the effects of yoga meditation intervention on mental health indicators in female college students.

The SEM analysis (Figure 2) illustrates that yoga meditation directly reduced anxiety (path coefficient = −0.52, *p* < 0.001), depression (path coefficient = −0.48, *p* < 0.001), and perceived stress (path coefficient = −0.46, *p* < 0.001). The findings suggest that yoga meditation effectively alleviated negative emotions and reduced subjective stress in the short term post-intervention. This direct effect supports meditation’s core function as an emotional management tool, especially in helping individuals cope with various psychological challenges of college life. Additionally, meditation also indirectly improved mental health by enhancing psychological resilience (path coefficient = 0.38, *p* < 0.001) and emotional regulation (path coefficient = 0.41, *p* < 0.001). As mediators, psychological resilience and emotional regulation play a crucial role in reducing anxiety, depression, and stress. Psychological resilience increased participants’ adaptability to adversity, helping them better manage life’s challenges and maintain a positive mindset despite stress and negative emotions. Emotional regulation, particularly through cognitive reappraisal, enabled individuals to manage and adjust emotional responses more effectively, mitigating the lasting impact of negative emotions on mental health. These indirect paths further promote long-term improvements in mental health by enhancing individuals’ psychological resilience and emotional regulation.

The pathway coefficients in the model illustrate the significance of these mediating effects: psychological resilience and emotional regulation contributed to the reduction of anxiety (path coefficient = −0.45), depression (path coefficient = −0.47), and perceived stress (path coefficient = −0.44), positively impacting mental health in the long term. These results indicate that the lasting effectiveness of meditation lies not only in short-term emotional relief but also in the long-term enhancement of emotional regulation and psychological resilience, leading to deeper improvements in mental health.

Model fitting indices suggested a good fit between the model and the data (CFI = 0.958, TLI = 0.942, RMSEA = 0.045), further supporting the hypothesis that meditation improves mental health through direct and indirect pathways. Specifically, the CFI value indicates that the model explains a substantial proportion of the variance in the data, while the RMSEA value confirms that the model’s fit error falls within an acceptable range. This model fit ensures that the path model reasonably predicts the multidimensional effects of meditation on mental health. Yoga meditation not only directly improves mental health by reducing anxiety, depression, and stress but also reinforces these effects by enhancing psychological resilience and emotional regulation.

### 3.4 Thematic analysis of yoga meditation experiences

#### 3.4.1 T1 phase: manifestation of initial psychological distress

At the initial phase of the meditation intervention, participants reported significant psychological distress, characterized by high levels of anxiety, stress, and emotional fluctuation. These issues primarily stemmed from academic pressures, uncertainties regarding career prospects, and complexities in social relationships, all of which posed substantial challenges to the mental health of female college students. Participants displayed a notable psychological burden, with some even experiencing somatic symptoms, such as insomnia, difficulty concentrating, and irritability.

Interviews and meditation journals from this early stage revealed feelings of helplessness and frustration when facing these psychological pressures. One participant mentioned, “As exams approach, it feels like the pressure crushes my shoulders. I constantly worry about not meeting expectations, and this anxiety haunts me every day.” Another participant described similar emotional fluctuations: “Thinking about the pressures of graduating, I often feel lost about the future. The more I think about it, the stronger my anxiety becomes, affecting my daily studies and life.” The qualitative data from this phase highlighted that female college students often lack effective emotional regulation skills when coping with the multifaceted pressures of academics, daily life, and future career planning. They tended to rely on emotion-focused stress coping strategies, such as avoiding problems or excessive worrying, rather than actively addressing issues. This reliance on emotion-focused stress coping strategies further exacerbated emotional fluctuations, forming a vicious cycle of stress and anxiety. It is noteworthy that while some participants held a degree of hope that meditation could serve as an effective means to relieve emotional stress, most remained skeptical before starting meditation. Some participants expressed uncertainty about the effectiveness of meditation, doubting whether they could truly benefit from simple relaxation exercises given their heightened anxiety. One participant admitted, “I know meditation might help, but I’m not sure I can stick with it, especially when I’m feeling anxious; it’s hard to calm down.”

The qualitative analysis aligned with the high initial scores of anxiety, depression, and stress in the quantitative data, showing a clear negative psychological state among participants in the T1 phase. A deeper analysis of psychological distress at this phase revealed that a prerequisite for the meditation intervention was the participants’ understanding and acceptance of meditation. Most participants demonstrated a high sensitivity to their emotional issues at this stage but lacked effective coping strategies. This emotional distress and cognitive dissonance were typical characteristics before starting the meditation intervention.

The qualitative analysis at T1 revealed the participants’ psychological state before the intervention and provided theoretical support for the effectiveness of subsequent meditation practices. The participants’ heightened sensitivity to stressors and limited emotional coping strategies suggests that meditation might help by enhancing self-awareness and emotional regulation skills, potentially breaking this negative cycle. The data from this stage laid a foundation for understanding the intervention mechanisms of meditation, particularly in how meditation may facilitate a transition from anxiety to inner peace by altering individuals’ perceptions and emotional responses to stress.

#### 3.4.2 T2 phase: emotional fluctuations during initial meditation experiences

In the mid-phase of the yoga meditation intervention (T2), participants began to show dynamic shifts in psychological states, moving from initial distress to early stages of emotional regulation. However, this transition was not linear and was accompanied by noticeable emotional fluctuations. Analysis of qualitative data from this phase indicated that although some participants reported initial positive effects from meditation, many still experienced recurrent anxiety and stress. Early experiences with meditation often intensified emotional instability, which is a phenomenon termed the “early meditation effect” in the study, where heightened self-awareness during meditation initially brought latent negative emotions to the surface. Several participants mentioned difficulties maintaining focus and dealing with strong emotional interference during the early meditation sessions. One participant described, “Every time I start meditating, my mind fills with all kinds of distractions, especially those that make me anxious. Even though I try to focus on my breath to relieve these feelings, the first few sessions made me feel even more anxious.” This feedback revealed that participants became more aware of the complexity of their inner emotions and underlying anxieties during meditation, making them more sensitive to these feelings. However, as meditation continued, some participants reported positive changes, particularly in heightened emotional awareness and management skills. One participant noted, “At first, I felt very anxious, but I noticed that each time I focused on my breathing, my emotions gradually calmed down. Although I’m not completely free of anxiety, I can feel my control over my emotions strengthening.” These responses suggested that meditation did not immediately eliminate negative emotions but guided participants to observe and accept their emotions gradually, fostering a growing awareness of emotional regulation.

During this phase, participants frequently reported experiencing “emotional emergence,” wherein previously suppressed emotions resurfaced during meditation, prompting them to confront these feelings. Some participants found it challenging to cope with the intensity of these emotions, occasionally experiencing brief moments of frustration. One participant noted, “There were times during meditation when I suddenly felt overwhelmed like all my stress came crashing down at once. Instead of relaxing, meditation seemed to make me even more sensitive.” This phenomenon illustrates that in the initial stages of meditation, participants may experience heightened emotional awareness, which can lead to temporary emotional fluctuations and discomfort.

Notably, despite the prevalence of emotional fluctuations in the T2 phase, many participants recognized this as a natural part of the meditation process. Some began to understand that meditation is not a quick fix for eliminating all negative emotions but rather a practice to help them face and accept these emotions with a calmer mindset. One participant wrote in her meditation journal, “Meditation helped me realize emotions themselves aren’t frightening; what’s scary is how I used to ignore them. Now, I’ve learned not to avoid them but to acknowledge their presence and gradually let them go.”

The qualitative analysis from the T2 phase reveals the complex psychological experience at the beginning of meditation practice, especially regarding emotional fluctuations and the development of self-awareness. While these fluctuations temporarily intensified participants’ feelings of anxiety, in the long term, this emotional emergence and the process of addressing it allowed practitioners to learn how to manage and regulate negative emotions gradually. These fluctuations are considered a necessary step in the meditation process, where deep self-reflection leads participants into a critical stage of emotional regulation. Findings from the T2 phase suggest that meditation is not merely a relaxation tool but a training process for emotional regulation, helping practitioners establish greater psychological resilience by gradually confronting emotional distress.

#### 3.4.3 T3 phase: transition from emotional regulation to inner peace

In the late phase of the meditation intervention (T3), participants exhibited a significant shift in their psychological state, with many reporting enhanced emotional regulation abilities and an increasing sense of inner peace. Analysis of qualitative data from this phase showed that meditation helped participants manage emotional fluctuations effectively and furthered the development of psychological resilience and self-awareness. Gradually, participants felt liberated from the grip of anxiety and stress, with a growing sense of tranquility. Numerous participants expressed positive experiences with meditation, especially in terms of their increased resilience and calmness in handling external pressures. One participant commented during an interview, “Meditation helped me understand that anxiety and stress are temporary states, not permanent issues. I’ve learned how to quickly adjust my mindset when stress arises, avoiding the negative cycle of anxiety that I used to fall into.” This shift in response to stress marked participants’ gradual establishment of effective emotional regulation mechanisms, enabling them to face challenges more objectively and calmly rather than being dominated by emotions.

It is worth noting that many participants reported experiencing a lasting sense of inner peace during this phase, which extended beyond meditation sessions into their daily lives. Participants found that they could apply meditation techniques in real-life situations, particularly when facing academic pressure, interpersonal conflicts and uncertainty about the future. One participant wrote in her meditation journal, “Now, when I feel stressed, I pause for a moment and focus on my breathing, and within a few minutes, my mind calms down. This yoga meditation helps me respond to daily pressures more rationally and calmly.” This sustained inner peace indicates that meditation is a short-term tool for emotional regulation and a long-term means of building psychological resources.

In the T3 phase, participants gradually transitioned from emotional regulation to self-acceptance and inner balance. Some participants noted that meditation taught them to accept themselves and the presence of negative emotions without struggling against them. One participant shared, “I used to be so hard on myself, always thinking I wasn’t doing well enough or meeting others’ expectations. But now I realize that emotions are a part of life, and accepting them rather than avoiding them makes me feel more at ease.” This self-acceptance helped participants reduce inner conflicts and strengthen their self-esteem and confidence, enhancing their mental health.

Moreover, the long-term effects of meditation on emotional regulation became more evident in the T3 phase. Participants observed that meditation heightened their emotional sensitivity, allowing them to recognize and manage negative emotions at their onset. One participant mentioned, “Through meditation, I can now sense when anxiety is starting, rather than noticing only when it peaks. I’ve learned to intervene early instead of letting it build up.” This feedback demonstrates that yoga meditation enables individuals to manage their emotions, proactively preventing further escalation.

The qualitative analysis from the T3 phase illustrates the transition from emotional regulation to inner peace, further supporting the effectiveness of meditation as an emotional management tool. Meditation goes beyond emotional regulation at this stage, facilitating a deeper state of inner peace and self-acceptance. This process reflects a gradual liberation from external pressures and internal anxiety, enhancing psychological resilience and emotional stability. Findings from this phase suggest that the long-term benefits of meditation lie in establishing a stable emotional regulation mechanism that reduces the influence of negative emotions. Additionally, meditation fosters acceptance of self and emotions, which, in turn, strengthens a sense of inner balance and supports mental health.

#### 3.4.4 T4 phase: lasting psychological impact post-meditation

In the follow-up stage after the end of the meditation intervention (T4), participants demonstrated a consolidation of their psychological state, reflecting the enduring impact of meditation. Qualitative data analysis revealed that meditation significantly affected emotional regulation and psychological resilience during the intervention and provided participants with a sustainable tool for managing emotions in their daily lives. Participants widely reported that meditation had lasting benefits for their long-term mental health, particularly in applying learned techniques when encountering new stressors. Many participants mentioned in interviews that, despite the end of the intervention, they continued to experience a sense of inner peace and emotional stability brought about by meditation. One participant shared, “Even though the program has ended, I’ve kept up my meditation habit. Whenever I feel anxious, I take a few minutes to meditate, and it helps me quickly distance myself from the stress.” Feedback from this phase indicated that participants maintained high levels of psychological resilience and emotional regulation after the intervention, particularly when facing complex academic demands and uncertainties about the future. One participant recalled, “During the end-of-term exam period after the intervention, I felt much less stressed because I had learned to manage my emotions. Through meditation, I no longer felt overly anxious about exams and approached them with a calmer mindset.” This feedback highlights how meditation practice improved participants’ ability to manage stress, enabling them to handle pressure situations more rationally and reduce anxiety’s impact.

In the T4 phase, participants not only reported the sustained impact of meditation on emotional regulation but also highlighted its long-term benefits for personal growth and self-identity. One participant wrote in her meditation journal, “Meditation has helped me understand myself better. I’ve started to recognize my true needs and no longer focus excessively on others’ opinions but instead center on my inner self. This change has made me more confident and at peace.” This increased self-awareness illustrates the significant effect of meditation in facilitating deep self-reflection and self-acceptance. Additionally, the long-term effects of meditation included an enhancement of emotional awareness. Participants noted that they became more attuned to early signs of emotional changes and could adjust their responses, preventing emotional escalation. In a follow-up interview, one participant stated, “I used only to recognize an issue when emotions became intense, but now I can sense shifts much earlier and immediately turn to meditation or other techniques to cope. This early awareness allows me to manage my mental state better.” This increased emotional awareness demonstrates that meditation helps participants address emotions in the moment and strengthens their ability to prevent emotional distress from escalating.

The qualitative analysis from the T4 phase further supports the long-term benefits of meditation intervention, revealing its lasting impact on participants’ mental health. Through meditation, participants acquired emotional regulation skills during the intervention and retained these psychological resources after the intervention, enabling them to maintain emotional balance and psychological resilience under new life pressures. Findings from this phase suggest that meditation’s effects extend beyond short-term relief from anxiety and stress, bringing more enduring improvements in mental health through heightened emotional awareness, self-acceptance, and psychological resilience. This sustained impact demonstrates the broad applicability of meditation as a mental health intervention, providing emotional support during the intervention period and long-term benefits for individuals after the intervention. The findings from the T4 phase further confirm meditation’s effectiveness in supporting long-term mental health, particularly in areas of emotional management, self-awareness, and psychological resilience, providing important theoretical support for promoting meditation as a psychological intervention with substantial empirical backing for its broader application.

### 3.5 Mechanisms underlying the positive psychological effects of yoga meditation on female college students

Quantitative research showed that yoga meditation has a significant positive effect on the mental health of female college students. In contrast, qualitative research revealed how it enhances self-awareness, regulates emotions, and improves stress management, helping female students achieve psychological balance and resilience when facing academic and life challenges. This mechanism can be examined from four perspectives: central neural systems, autonomic nervous system, emotional regulation, and long-term effects.

#### 3.5.1 Changes in the central neural system

Yoga meditation enhances neuroplasticity in the brain, modulating neural pathways associated with emotional regulation, self-awareness and cognitive control, helping individuals better cope with stress and emotional fluctuations. Studies have shown that regular meditation can significantly alter the structure and function of key brain regions, such as the prefrontal cortex, anterior cingulate cortex, insula, and hippocampus (Gotink et al., 2016). These areas are vital in self-awareness, emotion regulation, and cognitive processing. Increased cortical thickness in the prefrontal cortex is closely associated with improved attentional control, while activation in the prefrontal cortex aids in managing emotional conflicts and stress. Meditation also strengthens neural connectivity in these regions, enhancing individuals’ capacity for emotional regulation and enabling them to manage emotional responses more flexibly (Kass, 2015). Research indicates that meditation reduces amygdala activity, diminishing the intensity of responses to negative emotions. This change means that female college students practicing meditation can recover from emotions such as anxiety and fear more quickly, thereby minimizing their negative impact on daily life (Kass, 2015). Furthermore, meditation increases the activity of alpha and theta waves, helping the brain enter a relaxed and focused state, which enhances emotional stability and promotes inner peace (Magalhaes et al., 2018).

#### 3.5.2 Autonomic nervous system regulation

Meditation modulates the autonomic nervous system primarily by reducing sympathetic activity and increasing parasympathetic activity, thereby helping individuals cope better with stress and anxiety (Zaccaro et al., 2018). During meditation, heart rate and blood pressure decrease, and parasympathetic activity is enhanced, allowing the body to relax and recover. These physiological changes attenuate the hypothalamic–pituitary–adrenal (HPA) axis activity, reducing excessive stress responses and alleviating perceived stress among participants. Breath meditation, a key component of yoga meditation, consciously controls breathing, directly influencing heart rate variability and cardiac vagal tone (Zaccaro et al., 2018). Studies have shown that increased cardiac vagal tone is closely related to improved emotional regulation and psychological adaptability (Sarika and Ghosh, 2021). By enhancing vagal activity, meditation enables practitioners to manage stress better and quickly restore inner balance when facing negative emotions. This positive effect on the autonomic nervous system enhances the stress resilience of female college students and improves sleep quality, further promoting psychological health (Palek, 2020).

#### 3.5.3 Enhancing emotional regulation and psychological resilience

Emotional regulation is one of the core mechanisms through which meditation improves mental health. Through meditation, female college students learn to progress from emotional awareness to acceptance, effectively managing emotional fluctuations (Palek, 2020). Meditation facilitates the shift from a “doing” mode to a “being” mode, allowing participants to openly accept their current emotional experiences rather than avoid or suppress negative emotions. This open attitude helps them manage emotional fluctuations with a calm mindset, reducing the negative impact of emotions. Meditation also enhances metacognitive abilities, enabling practitioners to view emotions as transient mental events rather than an accurate reflection of reality. This cognitive shift allows female students to more flexibly regulate their emotional responses and reduce excessive reactions to negative emotions, thereby enhancing emotional regulation (Palek, 2020). With continued meditation, participants’ awareness and management of their emotions gradually improve, significantly increasing psychological resilience. Increased psychological resilience means that individuals are better equipped to face academic pressures and life challenges with a positive and composed mindset, reducing the impact of anxiety, depression, and other negative emotions (Schiweck et al., 2019).

#### 3.5.4 Long-term effects and sustained improvement

The long-term effects of meditation extend beyond short-term emotional regulation and stress relief. More importantly, it provides female college students with a sustainable tool for long-term emotion management. After 16 weeks of meditation practice, participants could maintain good emotional regulation skills even after the intervention ended and effectively apply meditation techniques when encountering new life stressors (Schiweck et al., 2019). This long-term effect indicates that meditation is a short-term psychological intervention and a long-lasting self-regulation strategy that helps individuals maintain inner peace and mental health after the intervention concludes.

## 4 Conclusion and recommendations

### 4.1 Conclusion

A systematic analysis of the effects of yoga meditation on the mental health of female college students found that meditation significantly improved anxiety, depression, perceived stress, and psychological resilience. Meditation had a positive impact on emotional regulation, self-awareness, and resilience. Over the 16-week intervention, participants gradually transitioned from severe psychological distress to inner peace and learned how to regulate emotions and enhance self-awareness, ultimately achieving emotional stability and increased psychological resilience. Yoga meditation effectively improved participants’ emotional management abilities by modulating the central neural system, autonomic nervous system and emotional regulation mechanisms. Its long-term effects and sustained improvements helped them approach academic and life pressures more calmly and confidently.

### 4.2 Recommendations

#### 4.2.1 Promote yoga meditation as a mental health intervention in higher education

Yoga meditation effectively alleviates anxiety and depressive symptoms arising from academic and life pressures in female college students. It is recommended that universities integrate yoga meditation courses into the mental health education system as an important intervention for promoting mental health. Universities can establish regular yoga meditation courses and provide meditation training workshops during extracurricular hours to help students understand the principles and techniques of meditation, thereby enhancing their mental health.

#### 4.2.2 Encourage the long-term maintenance of meditation habits

The positive effects of yoga meditation depend on sustained long-term practice, and the mental health benefits of meditation can continue beyond the intervention period. It is recommended that universities establish supportive mechanisms to help students develop lasting meditation habits. For instance, universities could develop dedicated meditation apps or collaborate with existing meditation platforms, using regular reminders, tracking practice times, and providing personalized meditation plans to help students stay motivated. Additionally, universities could encourage peer exchange through campus clubs or online sharing platforms to foster a supportive meditation community.

#### 4.2.3 Provide diversified and personalized meditation guidance

The psychological needs and stressors of individuals vary, and the effect of yoga meditation on emotional regulation differs from person to person. It is recommended that university counseling departments offer personalized meditation guidance to ensure that each student can fully benefit from meditation. Meditation modules can be designed based on specific issues such as anxiety, depression, or poor sleep quality. For students with high anxiety levels, breathing-focused and body scan techniques may be emphasized. For those struggling with insomnia, relaxation meditation before bedtime could be recommended. Such personalized meditation plans would better meet students’ needs and enhance the effectiveness of the intervention.

#### 4.2.4 Integrate mental health theory education with meditation practice and other psychological interventions

Mental health education is crucial for enhancing students’ overall psychological well-being. Offering elective courses related to meditation and psychology, which explore meditation’s mechanisms and its effects on the brain, would facilitate the integration of mental health theory education with meditation practice. Moreover, combining meditation with other psychological interventions, such as cognitive behavioral therapy (CBT), mindfulness training, and group counseling, would enhance the therapeutic effects. Meditation techniques could be used as auxiliary tools during CBT sessions to help students better regulate emotions and cope with negative thoughts during cognitive restructuring. In group counseling, group meditation could strengthen emotional connections among members and improve group atmosphere, thereby effectively reducing collective anxiety and stress while maximizing improvements in students’ mental health.

## Data Availability

The raw data supporting the conclusions of this article will be made available by the authors, without undue reservation.
